# 818. Optimizing Burn Dressing Procedures: The Impact of Staffing and Dressing Times

**DOI:** 10.1093/jbcr/irag033.257

**Published:** 2026-04-06

**Authors:** Jillian Lloyd

**Affiliations:** Orlando Regional Medical Center, Ocoee, FL

## Abstract

**Introduction:**

Burn dressing changes are complex, resource-intensive procedures requiring precise coordination among multidisciplinary care teams. The number and composition of staff present during these procedures may significantly influence workflow efficiency, procedure duration, and patient outcomes. Despite the critical nature of these interventions, our unit has not previously documented the relationship between staffing levels and dressing change duration.

Recent literature underscores the importance of adequate nurse staffing in burn care settings. It adds to a nurse’s workload in burn centers and increases mortality by 30%. This findings highlight the broader implications of staffing on care quality and patient safety.

The purpose of this project is to improve patient care, comfort, and outcomes while using staff and time more effectively.

**Methods:**

The Clinical Assistant Nurse Manager reviewed the duration of burn dressing changes and the number of staff members present. Dressing time was defined as the interval from initial wound exposure to the completion of new dressing application. Data was analyzed to identify correlations between staffing and procedure duration.

**Results:**

Data collected across multiple dressing changes revealed notable variation in procedure efficiency relative to team size. The trends suggest that both understaffing and overstaffing may contribute to prolonged dressing times. In contrast, procedures involving a balanced number of staff—typically 3 to 4 members depending on wound complexity—were associated with shorter and more streamlined dressing durations.

These findings align with broader research on workflow optimization in wound care. Studies have shown that well-defined team roles and streamlined workflows can significantly improve efficiency and reduce clinician burden. Moreover, excessive staffing may lead to role ambiguity and workflow congestion, while insufficient staffing can compromise safety and prolong care delivery.

**Conclusions:**

Documentation of burn dressing times in relation to staff presence is both feasible and valuable. Our findings provide actionable benchmarks for optimizing staffing models and procedural workflows in burn care settings.

**Applicability of Research to Practice:**

This study offers practical insights for nursing leadership and operational planning:

Staffing Allocation: Data-driven staffing models can help ensure optimal team size for burn dressing procedures.

Workflow Efficiency: Balanced staffing supports smoother task execution and reduces procedure time.

Patient Safety: Appropriate staffing minimizes risks associated with prolonged wound exposure and procedural delays.

Future research should explore the impact of team composition (e.g., skill mix, experience level) and role clarity on dressing change outcomes. Additionally, integrating workflow technologies and standardized protocols may further enhance efficiency and safety in burn care.

**Funding for the study:**

N/A.

